# Advancing usability of an influenza hemagglutinin virus-like particle vaccine expressing a chimeric cytokine

**DOI:** 10.1186/s12985-023-02076-1

**Published:** 2023-05-26

**Authors:** Toshifumi Imagawa, Youta Arasaki, Kenichi Maegawa, Shigeo Sugita, Kuniaki Nerome

**Affiliations:** 1 Nerome Institute of Biological Resources, Nago, Okinawa Japan; 2 Equine Research Institute, Japan Racing Association, Shimotsuke, Tochigi Japan

**Keywords:** Chimeric cytokine, Virus-like particle, Vaccine, Influenza virus

## Abstract

Vaccine efficacy of conventional influenza vaccines depend on the antigenic similarity between the selected vaccine strain and annual epidemic strain. Since the influenza virus evolves yearly, a vaccine which is independent from viral antigenic mutation is desired. We have developed chimeric cytokine (CC) and hemagglutinin (HA) incorporated virus-like particle (CCHA-VLP) as a universal influenza vaccine candidate. Using mouse models, it was shown that the vaccine provided broad-based protective activity against several types of human and avian influenza A viruses. In this report, nasal immunization and mixture form (CC- and HA-VLP) were tested to improve usability of this vaccine. Immunogenicity was evaluated by induction of IgG, IgA, and IFN-γ secreting cells. Protective activity was measured as mouse survival rate against lethal challenge with H1N1 and H5N1 viruses and against H3N2 virus by lung viral titer. Nasal immunization showed low immunogenicity and low protective efficacy, but the addition of a sesame oil adjuvant improved vaccine efficacy. Mixture form of CC- and HA-VLP showed comparable or higher vaccine efficacy when compared to the incorporated form, CCHA-VLP. These results contribute to improved usability, such as needle-less administration and easy HA subtypes alteration.

## Introduction

Influenza virus is one of the major pathogens of acute respiratory infection and can cause acute pneumonia. It is estimated that seasonal influenza results in 870,000 hospitalizations and 15,000 acute lower respiratory tract disease related deaths in children younger than 5 years of age, and 5.7 million adult hospitalizations [1, 2]. Antiviral drugs are widely used to reduce the severity of influenza symptoms and shorten the time of illness, however, vaccination is still the most effective countermeasure to control for mitigating the influenza epidemic. The current licensed influenza vaccines target the viral surface hemagglutinin (HA) protein which is highly antigenic. Between 2004 and 2020, vaccine effectiveness ranged from 10 to 60% in USA, and varied due to antigenic mismatch with circulating strains [3]. The influenza virus often evolves via antigenic mutation which confers immune-escape and low vaccine efficacy. Therefore, current research focuses on the development of a universal influenza vaccine that targets conserved viral regions and is effective against various influenza strains. Several universal influenza vaccine candidates have been developed using various strategies. For example, a matrix 2 (M2) protein-based vaccine was developed, as it is highly conserved across influenza subtypes, and induced an immune response against various types of influenza subtypes [4, 5]. In addition, inducing T cell immunity is an effective method to induce broad-based immunity [6]. Therefore, we focused on M2 peptide and interleukin (IL)-12 which stimulates T cell response, and constructed chimeric cytokine (CC) consisting of M2, neuraminidase (NA) stalk and IL-12 in previous study, then produced virus-like particles (VLPs) containing CC and HA proteins. The VLP vaccine efficacy was evaluated in mice and was shown to induce broad-based immunity against homologous heterologous influenza serotypes [7]. However, there were several VLP-related problems, such as difficulty adjusting for HA and CC quantity separately because these proteins were incorporated in same particle together. If the HA and CC proteins are not required to express on the surface of same VLP for vaccine efficacy, it is possible to adjust quantity of each protein separately, leading to improve the usability of the vaccine.

Most vaccines are administered via subcutaneous or intramuscular injection, however, it is estimated that one in four adults and two in three children fear injection [8]. Therefore, there is a need to identify alternate immunization routes, such as nasal or oral routes. Nasal immunization is suitable for influenza vaccine since it would be efficiently recognized by antigen presenting cells whereas via the oral route, the vaccine would be exposed to digestive enzymes in the stomach. It has been previously reported that mucosal immunity, such as nasal IgA, was induced by nasal influenza vaccine immunization [9]. In 2003, an intranasal live attenuated influenza vaccine was approved for use in the United States, and the effectiveness is comparable to that of the standard inactivated vaccine [10]. Because of these advantages, trying intranasal immunization would be benefitable for development of our VLP vaccine.

As mentioned above, intraperitoneal injection of our vaccine successfully induced broad-based immunity, in mice, against influenza A virus subtypes [7]. However, there was still room to improve the usability. In this study, we aimed to improve the usability of our developed vaccine and evaluated different immunization routes, such as intranasal administration. In addition, we evaluated immunization with the mixture of HA- and CC-VLPs.

## Methods

### Producing and purifying recombinant VLPs

The CC-, (Fukushima H5) FkH5-, CCFkH5-VLP were produced as described previously [7]. Briefly, FkH5-HA and CC coding nucleotide sequences were incorporated into the *Autographa californica* nuclear polyhedrosis virus (AcNPV) genome using the bac-to-bac baculovirus vector system. The FkH5 HA sequence was derived from the A/tufted duck/Fukushima/16/2011/(H5N1) strain (GenBank accession number: AB629698). Recombinant viruses, FkH5-AcNPV and CC-AcNPV were co-inoculated or separately infected into Eri-silkworm pupae, obtained from Dr. Kajiura in Shinshu university. Infected pupae were collected nine days post-infection. Pupae were homogenized and purified by ultracentrifugation and sucrose cushion centrifugation. The resultant VLP solution was used for immunization. HA protein was quantified using the hemagglutination test and CC was expressed as IL-12 which was quantified using the Lebis mouse IL-12 ELISA assay kit (Fuji film Wako, Osaka, Japan), according to the manufacture’s protocol.

### Hemagglutination test

Two-fold serial dilutions of the virus and VLP samples were performed with PBS as the diluent. Then, chicken red blood cell suspension (0.5%) was dropped into the sample, mixed, and incubated for 1 h. Following incubation, the HA titer was determined.

### Mouse immunization and virus challenge

Six week-old female BALB/c mice were purchased from SLC Japan. CCFkH5-VLP was administered to the mice three times on days 0, 7 and 14 with using three administration methods: (1) intraperitoneal route, (2) intranasal route and (3) intranasal route with a sesame oil adjuvant. In addition, to evaluate the necessity to incorporate CC and HA in single VLPs, the mixture of CC- and FkH5-VLPs was administered to mice via the intraperitoneal route. Each aliquot contained approximately 25,000 HA units and 50 ng of IL-12 protein in 200 µL for intraperitoneal injection or in 50 µL for intranasal immunization. Seven days following the third immunization, mice were infected with A/Puerto Rico/8/1934/H1N1 (PRH1), A/Aichi/2/1968/H3N2 (AiH3) or low pathogenic avian influenza (vaccine seed) viruses, RG-A/Barn Swallow/Hong Kong/1161/2010-A/PR/8/34 H5N1 [R] (6 + 2) (HKH5). The PRH1 and HKH5 infected mice were monitored for two weeks and their health condition and body weight were observed. The AiH3 infected group was euthanized four days post infection (dpi). Since low mortality was observed in this group, the lung tissue viral titer was measured using the plaque assay. Ten weeks-old BALB/c mice infected with PRH1, AiH3 or HKH5 virus served as the unvaccinated control groups and viral pathogenicity was evaluated. To analyze the induction of humoral and cell immunity, immunized mice were euthanized seven days post immunization and unvaccinated mice were used as control group. Serum, nasal and vaginal wash and spleen were collected from these mice to use for subsequent assays. Nasal and vaginal wash was obtained immediately after euthanasia by washing nasal cavity and vagina with 100 µL of PBS. Immunization and virus challenge experiments were performed with groups of five mice per each.

### Plaque forming assay

Confluent Madin-Darby Canine Kidney (MDCK) cells were inoculated with serial ten-fold dilutions of the sample serum in a multi-well plate and incubated for 1 h at 37 ℃. Agar (0.9%)-containing 1x MEM and trypsin was overlayed and infected cells were incubated for three days at 37 ℃ and 5% CO_2_. After fixation with 10% formalin, the agar was removed and the cells were stained with Coomassie brilliant blue (CBB). The number of plaques generated was counted visually and the viral titer was calculated as plaque forming units per volume (PFU)/mL.

### Enzyme-linked immunosorbent assay (ELISA)

Anti-FkH5 and anti-M2 IgA were evaluated using ELISA. The FkH5-VLP suspension was mixed with Triton-X100 (0.1%) and incubated for 5 min. FkH5 concentration was measured using the pierce BCA protein assay kit (Thermo Fisher Scientific, Waltham, USA), according to the manufacture’s protocol. Coating solutions were prepared with the abovementioned FkH5 solution, M2e peptide (GenScript, Piscataway, USA), and adjusted to 30 µg/mL and 5 µg/mL, respectively with 0.1 M carbonate-bicarbonate buffer (pH 9.5). The coating solutions (100 µL) were added into wells of a Nunc-Immuno plate II (Thermo Fisher Scientific), and incubated overnight at 4 ºC. The coating solution was removed and the plate wells were washed with PBS supplemented with 0.05% tween 20, and blocked with block ace (KAC, Kyoto, Japan) for 30 min at room temperature. Following a PBS wash, the tested sera were added into the wells and incubated for 2 h at room temperature. Plates were washed and coated in goat anti-mouse IgG (H + L) antibody, HRP conjugate (Proteintech group, Inc., Rosemont, USA) or Goat anti-mouse IgA-HRP (Southern Biotech, Birmingham, USA) in 1:10,000 dilution and incubated for 1 h at room temperature. Following washing, 1-step TMB ELISA substrate (Thermo Fisher Sientific) was used, according to the manufacture’s protocol. The optical density at 450 nm (OD_450_) of each well was measured with Multiskan Sky High Microplate Spectrophotometer (Thermo Fisher Scientific). Each group was compared based on relative OD_450_ which was calculated as OD_450_ of each sample was divided by the mean of unvaccinated control group.

### Mouse IFN-γ ELISpot assay

Splenocytes were prepared as previously described [7]. Briefly, collected spleen was homogenized and purified by centrifugation using NH_4_Cl as the hemolytic agent. The cells were fractionated with a 40 μm pore cell strainer and stored at -80 ℃. Collected mouse splenocytes were added at to plates at 10^5^ cells/well and stimulated with 16 HA titers of the H5N1 virus for 24 h. IFN-γ–secreting cells were detected using mouse IFN-γ Single-Color ELISpot (Cellular Technology Limited, Cleveland, USA), according to the manufacturer’s protocol. Spots were stained, counted, and calculated as the number of IFN-γ–secreting cells per million splenocytes. Samples that did not contain 10^5^ cells/well at incubation with antigen were excluded from analysis. Therefore, the number of data in each group was five (unvac, CCFkH5 IP, CC + FkH5 IP) and four (CCFkH5 IN, CCFkH5 + SO IN).

### Statistical analysis

To compare the ELISA and viral titer data across groups, statistical analysis was performed using Welch’s test and R software version 3.6.2 [11]. P-values were adjusted by Bonferroni method and a value less than 0.05 was considered statistically significant.

## Results

### Induction of IgG and IgA in serum and mucosal samples

**Fig. 1:**
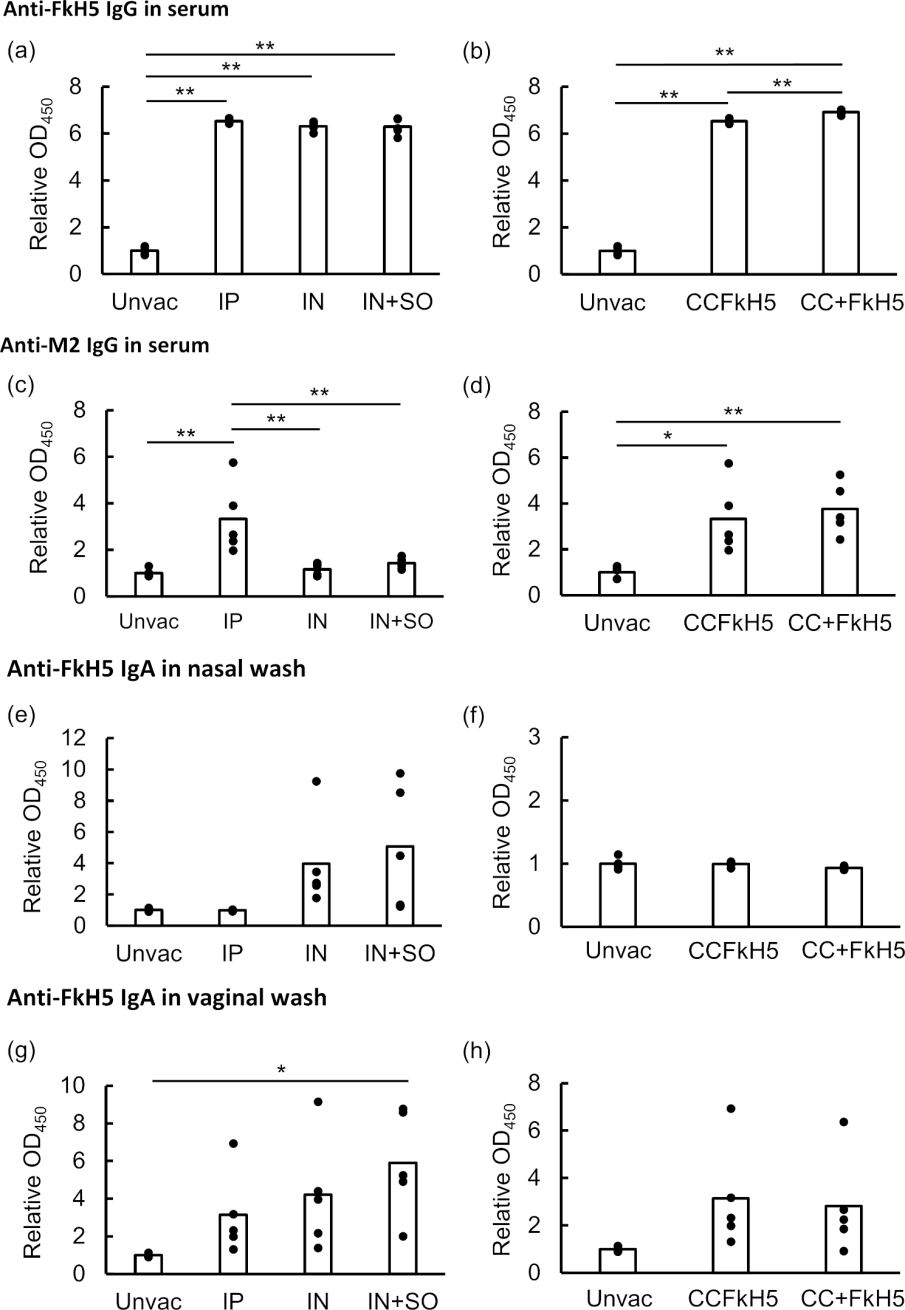
Induction of IgG and IgA in serum and mucosal samples. Six week-old BALB/c mice were immunized three times a week with CCFkH5-VLP containing 25,000 HA units and 50 ng of IL-12 protein by intraperitoneally (IP), intranasally (IN) or intranasally with sesame oil (SO), or mixture of CC- and FkH5-VLP intraperitoneally. The unvaccinated mouse group was indicated as “unvac”. Each group contains five mice. Serum, nasal wash and vaginal wash were collected one week after the third vaccination, and unvaccinated mice of same age were used as negative controls. Induction of IgG and IgA was evaluated using ELISA. The titer was expressed as relative optical density at 450 nm (OD_450_). Anti-FkH5 IgG in serum was compared across immunization routes (**a**) and between CCFkH5-VLP and mixture of CC- and FkH5-VLP (**b**). Anti-M2 IgG in serum was compared across immunization routes (**c**) and between CCFkH5-VLP and mixture of CC- and FkH5-VLP (**d**). Anti-FkH5 IgA in nasal wash was compared across immunization routes (**e**) and between CCFkH5-VLP and mixture of CC- and FkH5-VLP (**f**), and that in vaginal wash was compared across immunization routes (**g**) and between CCFkH5-VLP and mixture of CC- and FkH5-VLP (**h**). Statistical analysis was performed using pairwise Welch’s test and the p-value was adjusted using the Bonferroni method. The asterisk indicates statistical difference (*: p < 0.05, **: p < 0.01)

Anti-FkH5 IgG and anti-M2 IgG in serum and anti-FkH5 IgA in mucosal samples induced by each immunization method were measured by ELISA (Fig. 1). In the serum samples, intraperitoneal and intranasal methods significantly induced anti-FkH5 IgG (Fig. 1a), and a mixture of CC- and FkH5-VLPs immunization induced a higher anti-FkH5 IgG titer than that with CCFkH5-VLP (Fig. 1b). Anti-M2 IgG was significantly induced only in the intraperitoneal immunized groups (Fig. 1c), and there was no significant difference between the VLP-protein mixture and each individual VLP-protein (CC and FkH5HA) (Fig. 1d). Anti-FkH5 IgA in nasal wash was detected in intranasal immunization groups that were not induced by intraperitoneal immunization (Fig. 1e and f). Anti-FkH5 IgA was detected with several vaginal wash samples in both intraperitoneal and intranasal immunized groups, however, a significant difference was observed only in the group of intranasal immunization with sesame oil adjuvant (Fig. 1g and h). In this experiment, induction of anti-M2 IgG and anti-FkH5 IgA titers varied significantly among individuals.

### Induction of cell immunity

**Fig. 2:**
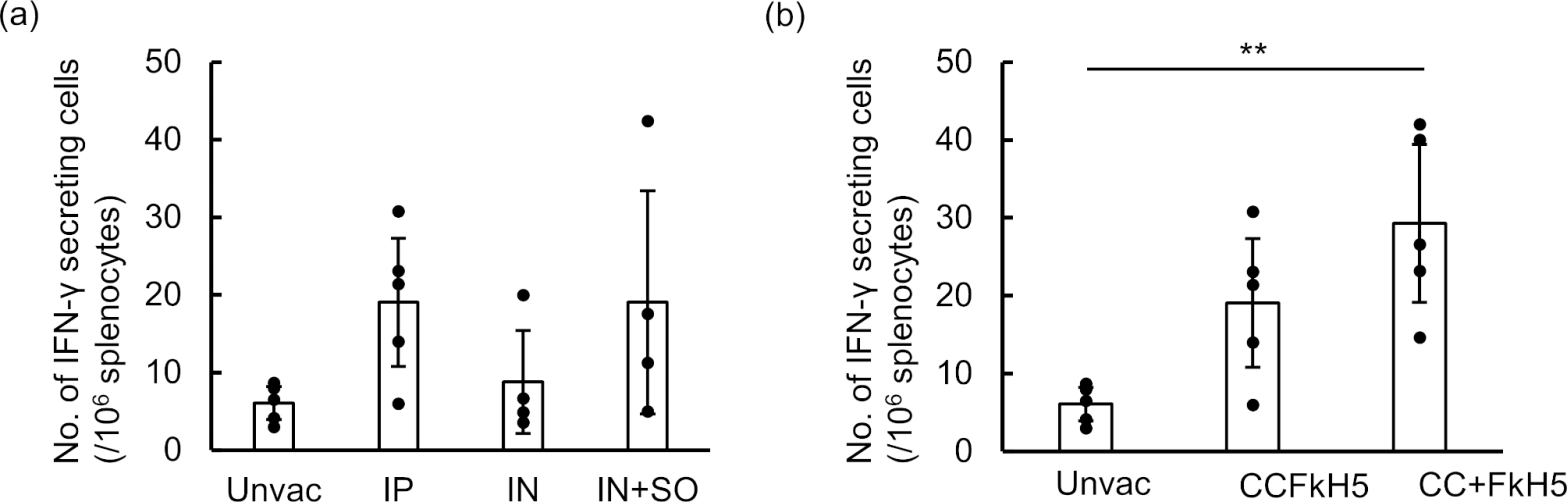
Detection of IFN-γ secreting cells in splenocytes. Spleen was concurrently collected with serum and mucosal sample collection from unvaccinated and immunized mice. IFN-γ secreting cells were measured with purified splenocytes by the mouse INF-γ ELISpot assay. HKH5 virus was used as the stimulating antigen. IFN-γ secreting cells in splenocytes were compared across immunization routes (**a**) and between CCFkH5-VLP and mixture of CC- and FkH5-VLP (**b**). Immunization methods were indicated as unvac: unvaccinated, IP: intraperitoneally, IN: intranasally and IN + SO: intranasally with sesame oil. Each group contains five mice, but IN and IN + SO groups contain four data. Error bars indicate 95% confidential intervals. Statistical analysis was performed using pairwise Welch’s test and the p-value was adjusted using the Bonferroni method, and the asterisk indicates statistical difference (**: p < 0.01)

To evaluate the induction of cell immunity by vaccination, IFN-γ secreting cells were measured using the IFN-γ ELISpot assay (Fig. 2). Although no significant results were observed by statistical analysis, IFN-γ secreting cells were induced in some immunized samples (Fig. 2a). These samples showed a confidence interval of greater than 95% in the unvaccinated group. In contrast, of the CC- and FkH5-VLPs combined protein immunized group displayed significant IFN-γ secreting cell induction (Fig. 2b).

### Comparison of protective activity among immunization methods

**Fig. 3:**
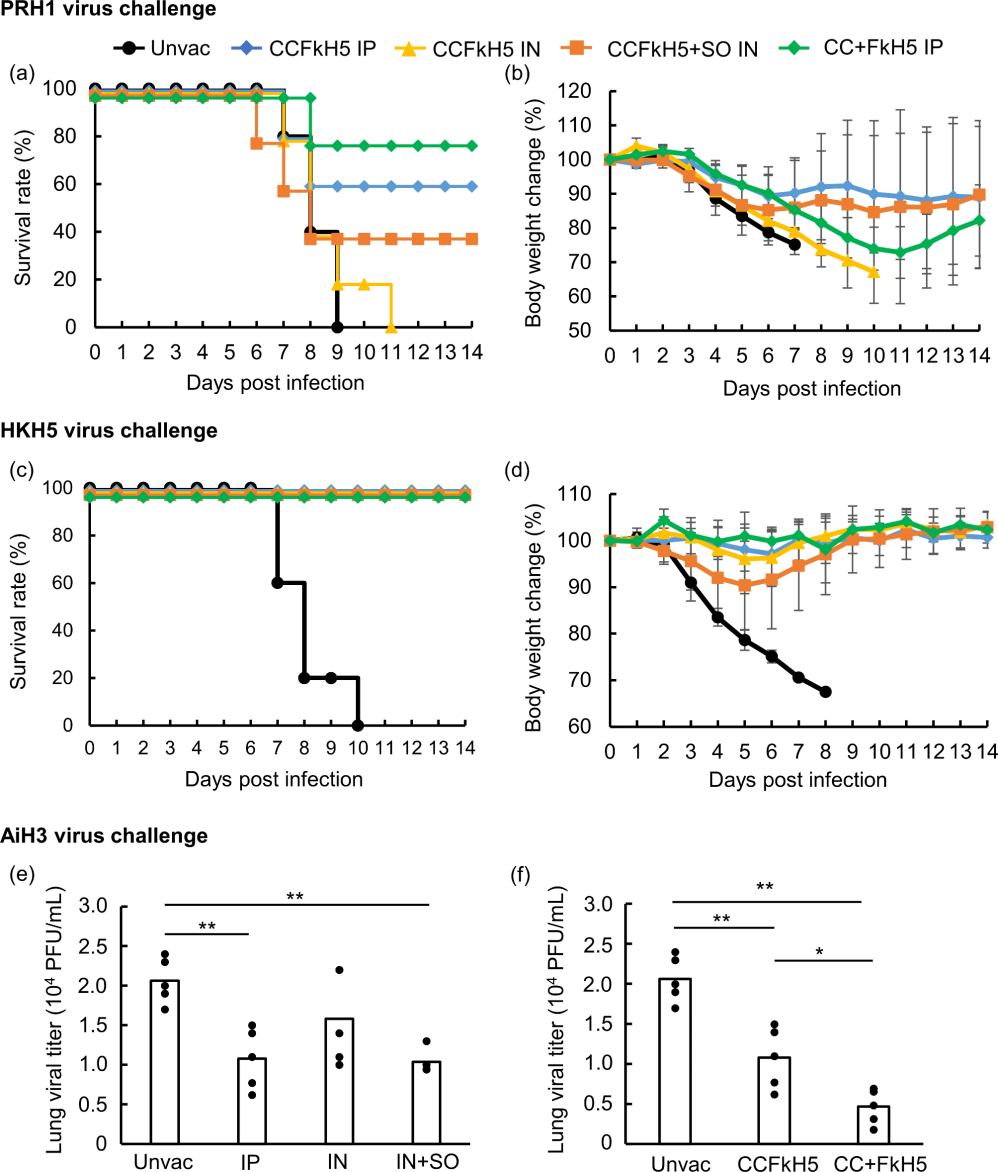
Protective activity of the CCFkH5-VLP vaccine against subtypes of influenza A virus. Unvaccinated and immunized mice were lethal challenged with PRH1 and HKH5 viruses one week after administration of the third vaccination. Survival rate and change in body weight were observed for 14 days. (**a**) Survival rates and (**b**) change in body weight in PRH1 virus challenge. (**c**) Survival rate and (**d**) change in body weight in HKH5 virus challenge. Protective activity against AiH3 virus was evaluated with viral titer in lung at four days post infection (4 dpi). The lung viral titer was compared across (**e**) immunization routes and (**f**) vaccine forms. Each group contains five mice and immunization methods were indicated as unvac: unvaccinated, IP: intraperitoneally, IN: intranasally and IN + SO: intranasally with sesame oil. Statistical analysis was performed using pairwise Welch’s test and the p-value was adjusted using the Bonferroni method, and the asterisk indicates statistical difference (*: p < 0.05, **: p < 0.01)

The protective activity of each immunization route and vaccine component was evaluated using PRH1 and HKH5 survival rate for lethal dose and lung plaque titer for AiH3 virus using BALB/c mice (Fig. 3). Although nasal immunization with CCFkH5-VLP did not protect mice from lethal PRH1 virus infection, survival rates improved with CCFkH5-VLP intranasal immunization with sesame oil adjuvant, CCFkH5-VLP intraperitoneal immunization, and CC-VLP + FkH5-VLP intraperitoneal immunization by 40%, 60% and 80%, respectively (Fig. 3a). In all immunization groups, mean body weight of mice was reduced by at least 10% (Fig. 3b). The highest survival rate was observed in the CC + FkH5 IP group, however, three of the four mice that survived had a significantly lower body weight. All immunized groups showed 100% survival rates against lethal HKH5 virus (Fig. 3c). In addition, the mean body weight loss across all immunized groups were less than 10% (Fig. 3d). Lung plaque titers were compared across immunization groups, at four days post AiH3 virus infection, and CCFkH5-VLP intraperitoneal immunization and CCFkH5-VLP with sesame oil by intranasal immunization significantly decreased viral titer (Fig. 3e). There was no significant difference between these two administration methods. Compared to the CCFkH5-VLP immunized group, a significantly lower viral titer was observed in the CC- and FkH5-group (Fig. 3f).

## Discussion

In this study, we evaluated the efficacy of intranasal CCHA-VLP vaccine administration. In addition, we evaluated the efficacy of CC- and HA-VLP. Initially, nasal CCFkH5-VLP immunization conferred a relatively low survival rate in the presence of lethal challenge with PRH1 virus. However, it induced mucosal IgA against the FkH5-VLP antigen compared with intraperitoneal immunization group. It implied that nasal immunization succeeded to induce mucosal immunity. The addition of a sesame oil adjuvant improved the survival rate of the nasal immunized group in the PRH1 virus challenge. Selection of a more appropriate adjuvant may improve CCFkH5-VLP vaccine efficacy via the nasal immunization route.

The sesame oil adjuvant group displayed a higher survival rate. Although no significant difference was observed, the sesame oil adjuvant improved vaccine efficacy as indicated by induction of antibodies and IFN-γ secreting cells. However, compared to intraperitoneal immunization, the lower PRH1 virus survival rates were observed in both nasal immunization groups which may be attributed to low anti-M2 IgG in serum. In agreement with this, in this study, it was observed that intranasal immunization induced a lower M2 IgG titer than intraperitoneal immunization. Similar results were observed in a study that compared intranasal and intraperitoneal immunization routes using M2 and HBV core fusion protein, however, higher protective activity against lethal infection was observed in the intranasal immunization [12]. Some mice of CCFkH5 with sesame oil intranasal immunized group showed large weight loss in especially HKH5 virus challenge. It was possibility that these mice were not successfully intranasally administrated with the vaccine because of viscosity of sesame oil, although immunological test was not performed for the challenged mice. As evidence, for example, the standard deviation of serum anti-FkH5-IgG titer which was less affected by sample collection technique than mucosal samples was larger than other immunized groups. Increasing the amount of CC and selecting a more effective adjuvant may improve the induction of anti-M2 IgG and the protective activity against heterotypic influenza virus by intranasal immunization.

CC and HA protein was added to the same VLP and its efficacy evaluated. Compared to CCFkH5-VLP immunization, CC- and FkH5-VLP immunization induced a higher quantity of in serum anti-FkH5 IgG and was associated with a higher PRH1 survival rate. However, the induction of anti-M2 IgG and anti-FkH5 IgA in mucosal samples were not significant between these groups. Considering these results, CC and HA protein combination does not provide higher efficacy and therefore it is not necessary to incorporate both proteins into the same VLP in a standardized manner. Instead, this result contributes to increased CC-VLP usability which can be combined with variable HA-VLP altered to match circulating strains. Using high priority subtypes, such as H1 and H3 as included HA, may provide maximum protective activity against homologous subtype and induce broad-based immunity against various serotypes. Since the combination vaccine can be prepared simply, it allows for easy CC/HA and HA subtype proportion alteration.

There were several limitations in this study. Firstly, large variety of IgA titers in mucosal samples was probably because of not only success rate of immunization but the disparity in sampling quantity obtained. Consequently, it was difficult to detect statistically significant differences between groups. However, anti-FkH5 IgA was induced in many samples. Second, although the amount of HA protein in vaccines was adjusted using an HA test, protein quantity may have differed by forms of incorporated VLP and mixture of HA- and CC-VLPs, because the number of HA protein contained in a single particle is probably different between CCFkH5-VLP and FkH5-VLP. This may have contributed to the differences in observed vaccine efficacy between CCfkH5-VLP and CC-+FkH5-VLPs immunized groups. Therefore, the incorporated vaccine was not superior, however, it successfully induced humoral and cell immunity comparable to the other vaccines.

Our vaccine showed 60–80% survival rate against heterologous H1N1 influenza virus in mice by intraperitoneal immunization. However, published universal influenza vaccine candidates, such as NA targeting vaccine and chimeric protein vaccine which consisted of M2 and HA stalks, showed excellent broad-based immunity against various influenza virus strains [5, 13]. A universal flu vaccine candidate, consisting of chimeric HA protein, underwent a phase I trial and promising results were obtained [14]. Our vaccine does not offer universal influenza vaccine efficacy. Nevertheless, developing vaccines possessing different immunization mechanisms is beneficial to counter measure against virus evolution and immune evasion. In this report, we have tested needle-less vaccination using HA- and CC-VLPs, to improve the usability of our vaccine in development. We plan to advance the chimeric cytokine vaccine to improve efficacy.

## Data Availability

Raw data used for drawing graphs and statistical analysis was available from the corresponding author on reasonable request.

## List of abbreviations

AcNPV: *Autographa californica* nuclear polyhedrosis virus
CC: chimeric cytokine
FkH5: Fukushima H5
HA: hemagglutinin
IL-12: interleukin-12
M2: matrix 2
VLP: Virus-like particle
